# Malaria outbreak investigation in Tanquae Abergelle district, Tigray region of Ethiopia: a case–control study

**DOI:** 10.1186/s13104-019-4680-7

**Published:** 2019-10-04

**Authors:** Kissanet Tesfay, Belete Assefa, Alefech Addisu

**Affiliations:** 1 Department of Epidemiology, Mekelle University College of Health Science School of Public Health, Mekelle, Ethiopia; 2 Department of Health System Management, Mekelle University College of Health Science School of Public Health, Mekelle, Ethiopia

**Keywords:** Case–control, Malaria, Ethiopia

## Abstract

**Objective:**

We investigated this outbreak to describe the magnitude and associated risk factors due to the malaria outbreak in Tanquae Abergelle district, Tigray, Ethiopia, in 2017.

**Result:**

Case fatality rate of this study was zero. Among the 62 cases and 124 controls, the presence of mosquito breeding sites [OR = 6.56 CI (2.09–20.58) *P* value = 0.001], sleeping outside a home [OR = 5.06 CI (1.75–14.61) P-value = 0.003] and having unscreened window [OR = 14.89 CI (1.87–118.25) P-value = 0.011] were associated with illness in multivariate analysis.

## Introduction

In 2017, globally malaria caused an estimated number of 219 million cases in 87 countries, and 435,000 deaths [1]. Around 90% of malaria deaths in 2016 took place in the WHO African Region [2].

Malaria is widely distributed in tropical and subtropical countries like Sub-Saharan Africa region because of consistent high humidity and high temperature. In 2015, globally Sub-Saharan African region accounts for 76% cases and 75% of malaria deaths [3]. In Ethiopia, around 60% of the populations live in malaria-prone areas, and 68% of the country’s landmass is favorable for malaria transmission. Malaria in Ethiopia has an unusual transmission pattern and large scale epidemics happen every 5–8 years. Malaria is the leading cause of morbidity and mortality in Ethiopia [4].

In the year 2014/2015 Ethiopia had a total number of 2,174,707 malaria cases. About 85.9% were confirmed either by microscopy or rapid diagnostic test (RDT). Of those, 63.7% were positive for *Plasmodium falciparum* [4].

In 2014/15 all malaria cases diagnosed in the Tigray region were 302,136. Among that, 86.8% were confirmed either by RDT or using microscopy. Of those, 70.2% were caused by *Plasmodium falciparum* [5].

Investigation of malaria outbreak is used to determine the specific cause or causes of the outbreak at the earliest time. And it is used to take appropriate measures and prevent future occurrence of malaria outbreaks. This would contribute to the national malaria elimination strategy by 2030. Therefore, the aim of this study is to describe the magnitude and risk factors associated with malaria outbreak in Tanquae Abergelle district, Tigray, Ethiopia, in 2017.

## Main text

### Methods

#### Study area

Tanquea Abergelle is one of the districts found in the central zone of Tigray region, Ethiopia. It is bounded on south and west by Amhara Region, on west Tekeze river separating it with Amhara region, on north Kola Temben district, on east Deguea Temben district and in the southeast Southeastern zone of Tigray region.

#### Study period

The study was conducted from September 8 to October 18, 2017.

### Study design

Descriptive epidemiology: During this outbreak, confirmed malaria case was an acute febrile illness with blood smear positive for malaria. Due to the incompleteness of the 2011–2016 malaria data, 2016s weekly malaria cases report was used to set the malaria epidemic threshold level. By doubling weekly data of 2016 and comparing it with the similar week of the year 2017 [6]. Data on malaria cases and deaths were obtained from Yechella primary hospital. This outbreak was described by age, sex, kebele, health facility, week, month and year. Similarly, slide positivity rate, attack rate, and the case fatality rate was calculated.

Analytical epidemiology: we conducted an unmatched case–control study in 1:2 ratio basis to identify risk factors associated with the disease from September 29 to October 18, 2017. Controls were selected from the community. Controls were defined as having no malaria signs and symptoms for the last 3 months and who did not have malaria by RDT during the outbreak period. During this investigation, a structured questionnaire was developed and adapted from different kinds of literature to assess risk factors for malaria. This includes data on patient age, sex, residence, family size, sleeping and staying area during the night, use of insecticide bed net, indoor residual spray, and presence of stagnant water or any other mosquito breeding area. The significance of risk factors for the outbreak was determined through multivariate analysis by calculating Odds Ratio (OR) and 95% confidence interval (CI).

#### Inclusion criteria

All laboratory-confirmed malaria cases of residents of Tanquae Abergelle attending Yechella primary hospital during the study period were included.

#### Exclusion criteria

Those severely ill and who were unconscious during the study period.

### Sample size determination

We calculated the sample size using the statistical software calculation of Epi-info taking the power of 80%, odds ratio of 0.3 for ‘sleeping under ITNs, percentage of exposed controls of 87.4%, and case to control the ratio of 1:2. The total sample size yields 170. With a 10% of non-response rate, our sample size was 186, with 62 cases and 124 controls [7].

#### Laboratory method

Thick and thin smears with a 100 × oil immersion microscopy was conducted by laboratory technicians of Yechella primary hospital.

#### Environmental assessment

In addition to interviews, environmental assessment of the presence of mosquito breeding sites of cases and controls in the radius of 500 meters near to their home was conducted. These include unprotected surface water, open deep well, solid and liquid waste collection and disposal facility. Similarly, observation of these potential mosquito breeding sites and the presence of Anopheles larvae in stagnant water were conducted.

#### Data collection

Data were collected using interviewer-administered a structured questionnaire.

#### Data processing and analysis

Data were entered and analyzed using SPSS software version 22.

#### Data quality control

Three days of training was given to data collectors on the data collection questionnaire. We used line list for describing malaria cases in terms of time, place and person. Data completeness was checked before analysis.

### Case definitions

Suspected: Patient with fever or history of fever in the last 48 h and lives in malaria-endemic areas or has a history of travel within the past 30 days to malaria-endemic areas.

Probable: Any person with fever and one or more of major sign such as headache, rigor, back pain, chills, sweats, myalgia, nausea, and vomiting diagnosed clinically as malaria [6].

### Result

#### Description of malaria by the person

From a total of 1300 suspected malaria cases, 694 (53.4%) were females. The median age of suspected malaria cases were 27 with interquartile range (IQR) of (19.40). The proportion of malaria cases was higher in females than in males.

The overall positivity of this outbreak was 876 (67.4%). Females had malaria positivity rate (PR) of 462 (66.5%). The age group of 5–14 had highest positivity 166 (76.8%), followed by 15–44 years of age 420 (67.3%) (Table 1).

**Table 1:** Distribution of malaria cases by age, sex and *Plasmodium species* in Tanquae Abergelle, 2017

Characteristics	Total tested (microscopy)	Total positive	Positivity rate (%)	*Plasmodium species*		
				*Plasmosdium falciprum* N (%)	*Plasmodium vivax* N (%)	Mixed N (%)
Sex						
Male	606	414	68.3	405 (66.8%)	9 (1.5%)	2 (0.3%)
Female	694	462	66.5	451 (64.9%)	11 (1.6%)	0
Age group						
0–4	337	213	63.2	209 (62%)	4 (1.2%)	1 (0.2%)
5–14	216	166	76.8	165 (76.4%)	1 (0.4%)	1 (0.4%)
15–44	624	420	67.3	410 (65.7%)	10 (1.6%)	0
> 44	123	77	62.6	72 (58.5%)	5 (4.1%)	0
Total	1300	876	67.4	856	20	2

The malaria attack rate of the catchment population was 33.1 per 1000 population. The case fatality rate of the catchment population was zero. The highest attack rate was among the age of 0–4 accounting 55.1 per 1000 population. Females had an attack rate of 34.2 per 1000 population (Table 2).

**Table 2:** Attack Rates and case fatality rates of malaria outbreak in Tanquae Abergelle district, Tigray, Ethiopia, 2017

Variables	Population	No of cases	No of deaths	Attack rate*1000	CFR (%)
Age group					
0–4	3864.6	213	0	55.1	0
5–14	7710.7	166	0	21.5	0
> 15	14912.7	497	0	33.3	0
Sex					
Male	12,979	414	0	31.9	0
Female	13,509	462	0	34.2	0
Total	26488	876	0	33.1	0

#### Description of malaria by place

Among the total of 1300 malaria suspected cases, 1024 (78.8%) were from Mearey kebelle.

#### Description of malaria by time

Tanquae Abergelle malaria outbreak had started weeks back before the outbreak was detected by field epidemiology residents in week 37. Malaria has an increasing trend during the study time. It had passed the threshold in all WHO epidemiologic week 28 to week 42.

**Fig. 1:**
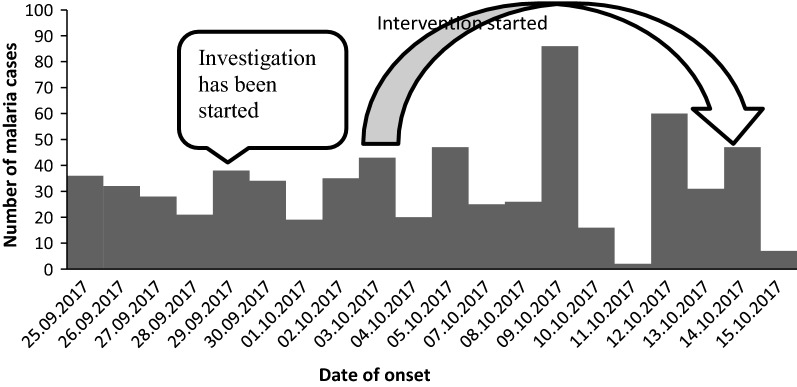
Epi curve showing of malaria outbreak in Tanquae Abergelle district, Tigray, Ethiopia, 2017

The investigation team departed to investigate this outbreak on 29.09.2017. Interventions were held starting from the time of the investigation (Fig. 1).

#### Risk factors analysis

Of the 62 case-patient 29 (46.8%), and 69 (55.6%) of the 124 controls were males with a response rate of 100%. The median ages of cases were 19 with IQR of (10.28) and controls were 30 with IQR of (22.44) (Additional file 1: Table S1).

In multivariate analysis the risk factors of sleeping outside home [AOR = 5.06 (CI 1.75–14.61) P-value of 0.003], the presence of mosquito breeding site around home [AOR = 6.56 (CI 2.09–20.58) P-value of 0.001], and having unscreened window [AOR = 14.89 (CI 1.87–118.25) P-value of 0.011] were associated with malaria.

#### Environmental observations

In our observation, there were multiple stagnant waters which could be potential mosquito breeding sites. There were also visible larvas in the stagnant waters especially in the Gerea kebelle of Tanquae Abergelle.

## Discussion

In the district, there were multiple mosquito breeding sites identified which might be a source of the outbreak. Moreover, indoor residual spraying of houses in affected kebeles with deltamethrin was not performed timely. Spraying in some rural kebelles had started after the outbreak had begun but didn’t involve the district town Yechella.

According to this case–control study, malaria was prevalent in females than males, an identified risk factor for malaria was the presence of mosquito breeding sites around home or vicinity, sleeping outside the home and having an unscreened window.

This study shows that malaria was more prevalent in females than in males. It was different from the finding of studies done in Oromia, and Gedio zone of Ethiopia [8, 9]. This might be due to improper use of ITNs, not giving priority to women’s to use ITN at home and spending more time outdoors during evening performing household chores.

Our study finding of sleeping outside the home was significantly associated with malaria. This was consistent with studies done in west Begal, India, and Swaziland [10, 11]. This might be due to the hot weather. During dry season people prefer to sleep outside in order to get fresh air and reduce heat. And this could make it difficult to use ITN’s while sleeping outside.

In our study, the finding of the presence of a mosquito breeding site around home or vicinity was significantly associated with malaria. This was in agreement with studies done in the Laelay Adeyabo, and Gedeo zone of Ethiopia, Tanzania, and Zimbabwe [9, 12–14]. However study done in Hadya zone, Ethiopia has no significant association between mosquito breeding site and malaria [15]. The difference with the study done in Hadya could be due to the far distance of the mosquito breeding site from the households.

The finding of having an unscreened window as a risk for malaria was in agreement with a study done in Equatorial Guinea [16].

## Conclusion

In Tanquae Abergelle district 15–44 years of age group and females were more affected by malaria. Malaria transmission had an increasing trend. There was a significant association of malaria with the presence of mosquito breeding sites around home or vicinity, sleeping outside a home and having an unscreened window. We would like to recommend the district health office to mobilize residents to avoid potential places of mosquito breeding site and to conduct regular indoor residual spray.

## Limitations

The selected disease-free controls might be in the incubation period for developing malaria.

## Supplementary information

**Additional file 1: Table S1.** Bi-variate analysis related to malaria outbreak in Tanquae Abergelle district, Tigray, Ethiopia, 2017.

## Data Availability

The datasets used and/or analyzed during the current study are available from the corresponding author on request.

## Abbreviations

AOR: Adjusted Odds Ratio
CI: confidence interval
CFR: case fatality rate
COR: Crude Odds Ratio
HEWs: Health Extension Workers
IQR: interquartile range
ITN: insecticide treated bed nets
OR: Odds Ratio
PF: *Plasmodium falciparum*
PR: positivity rate
PV: *Plasmodium vivax*
RDT: Rapid Diagnostic Test
TRHB: Tigray Regional Health Bureau
WHO: World Health Organization
