# Supplementary material for: Malaria outbreak investigation in Tanquae Abergelle district, Tigray region of Ethiopia: a case–control study
Source: BMC Res Notes. 2019 Oct 4;12:645. doi: 10.1186/s13104-019-4680-7 (PMC6778373; doi:10.1186/s13104-019-4680-7)
Supplement: Supplementary file 1 — Additional file 1: Table S1. Bi-variate analysis related to malaria outbreak in Tanquae Abergelle district, Tigray, Ethiopia, 2017. [file 13104_2019_4680_MOESM1_ESM.docx]

Table S1: Bi-variate analysis related to malaria outbreak in Tanquae Abergelle district, Tigray, Ethiopia, 2017

| Sr.no | Characteristics | | Case(N=62) | Control(N=124) | COR | 95% CI | P-value |
| --- | --- | --- | --- | --- | --- | --- | --- |
| 1 | Sleeping place | Inside | 48 | 75 | 1.56 | 1 | 0.016 |
|  |  | **Outside** | **14** | **49** | **2.24** | **1.12-4.49** | **0.023** |
| 2 | Do you stay outside? | No | 14 | 53 | 3.79 | 1 | 0.000 |
|  |  | **Yes** | **48** | **71** | **0.39** | **0.19-0.78** | **0.008** |
| 3 | Is there malaria ill person in your home? | No | 52 | 102 | 1.96 | 1 | 0.000 |
|  |  | Yes | 10 | 22 | 1.12 | 0.49-2.54 | 0.784 |
| 4 | Do you have Bed nets at home? | No | 20 | 60 | 3.00 | 1 | 0.000 |
|  |  | **Yes** | **42** | **64** | **0.51** | **0.27-0.96** | **0.038** |
| 5 | How often do you use Bed nets? | Never | 19 | 55 | 2.65 | 1 | 0.000 |
|  |  | Sometimes | 20 | 8 | 0.15 | 0.06-0.39 | 0.000 |
|  |  | Always | 23 | 61 | 1.09 | 0.54-2.22 | 0.809 |
| 6 | Do you give priority to mother and children to sleep in bed net? | No | 16 | 55 | 3.44 | 1 | 0.000 |
|  |  | **Yes** | **46** | **69** | **0.44** | **0.22-0.85** | **0.015** |
| 7 | Was deltamethrine sprayed this year? | No | 56 | 103 | 1.84 | 1 | 0.000 |
|  |  | Yes | 6 | 21 | 1.90 | 0.73-4.99 | 0.191 |
| 8 | Is there any of artificial water-holding containers close to your homes? | No | 18 | 93 | 5.17 | 1 | 0.000 |
|  |  | Yes | 44 | 31 | 0.14 | 0.07-0.27 | 0.000 |
| 9 | Presence of mosquito breeding sites around the home or vicinity? | No | 43 | 66 | 1.53 | 1 | 0.029 |
|  |  | **Yes** | **19** | **58** | **1.99** | **1.04-3.79** | **0.037** |
| 10 | Type of window? | Screened | 15 | 2 | 0.13 | 1 | 0.007 |
|  |  | **Unscreened** | **47** | **122** | **19.47** | **4.29-88.42** | **0.000** |
| 11 | Presence of unprotected irrigation around home? | No | 44 | 68 | 2.01 | 1 | 0.036 |
|  |  | **Yes** | **18** | **56** | **1.54** | **1.05-3.87** | **0.024** |
| 12 | Presence of tick grass around home? | No | 26 | 67 | 2.58 | 1 | 0.000 |
|  |  | Yes | 36 | 57 | 0.61 | 0.33-1.14 | 0.121 |
| 13 | Do you know sign and symptoms of malaria? | No | 23 | 71 | 3.09 | 1 | 0.000 |
|  |  | **Yes** | **39** | **53** | **0.44** | **0.23-0.82** | **0.010** |
| 14 | Do you know way of malaria transmission and prevention? | No | 19 | 69 | 3.63 | 1 | 0.000 |
|  |  | **Yes** | **43** | **55** | **0.35** | **0.18-0.67** | **0.002** |
